# Little needle-scalpel for piriformis syndrome: A protocol for systematic review and meta-analysis

**DOI:** 10.1097/MD.0000000000025242

**Published:** 2021-03-26

**Authors:** Qingyuan Zhu, Siyuan Zhu, Jun Xiong, Lunbin Lu, Jun Chen, Zhiying Zhong, Genhua Tang

**Affiliations:** a Nanchang Hongdu Hospital of Traditional Chinese Medicine; b Jiangxi University of Traditional Chinese Medicine; c The Affiliated Hospital of Jiangxi University of Traditional Chinese Medicine, Nanchang, PR China.

**Keywords:** acupotomy, Little needle-scalpel, meta-analysis, piriformis syndrome, protocol, systematic review

## Abstract

**Background:**

Piriformis syndrome (PS) is a condition in which the sciatic nerve is compressed when passing through the inferior mouth of the piriformis muscle, mainly caused by pain in one hip and leg. In severe cases, patients may experience severe buttock and lower limb pain, discomfort, difficulty walking, and claudication. It is estimated that the annual incidence of low back pain and sciatica is about 40 million cases, and the annual incidence of piriformis syndrome is about 2.4 million cases. The aim of this systematic review is to assess the effectiveness and safety of Little needle-scalpel therapy for Piriformis syndrome.

**Methods:**

Two reviewers will electronically search the following databases: the Cochrane Central Register of Controlled Trials (CENTRAL); PubMed; EMBASE; China National Knowledge Infrastructure (CNKI); Chinese Biomedical Literature Database (CBM); Chinese Scientific Journal Database (VIP database); and Wan-Fang Database from the inception, without restriction of publication status and languages. Additional searching including researches in progress, the reference lists, and the citation lists of identified publications. Study selection, data extraction, and assessment of study quality will be performed independently by 2 reviewers. If it is appropriate for a meta-analysis, RevMan 5.4 statistical software will be used; otherwise, a descriptive analysis will be conducted. Data will be synthesized by either the fixed-effects or random-effects model according to a heterogeneity test. The results will be presented as risk ratio (RR) with 95% confidence intervals (CIs) for dichotomous data and weight mean difference (WMD) or standard mean difference (SMD) 95% CIs for continuous data.

**Results:**

This study will provide a comprehensive review of the available evidence for the treatment of Little needle-scalpel with piriformis syndrome.

**Conclusions:**

The conclusions of our study will provide an evidence to judge whether Little needle-scalpel is an effective and safe intervention for patients with piriformis syndrome.

**Ethics and dissemination:**

This systematic review will be disseminated in a peer-reviewed journal or presented at relevant conferences. It is not necessary for a formal ethical approval because the data are not individualized.

**Trial registration number:**

INPLASY2020110092.

## 1 Introduction

### 1.1 Description of the condition

Piriformis syndrome is a common and frequently-occurring disease in orthopedics clinics and rehabilitation clinics, and is difficult to treat.^[1]^ Its clinical manifestations are mainly hip and leg pain and movement disorders. Mostly due to the anatomical variation of the piriformis, trauma, and other factors that cause the piriformis swelling, exudation, adhesion and contracture, and then compression of the sciatic nerve.^[2]^ 0.3% to 6% of people suffering from low back pain and sciatica may be related to piriformis syndrome. It is estimated that the annual incidence of low back pain and sciatica is about 40 million cases, and the annual incidence of piriformis syndrome is about 2.4 million cases. The majority of patients with piriformis syndrome are middle-aged patients, and the male to female ratio is about 1:6.^[3]^ The sciatic nerve is adjacent to the piriformis muscle, which is the external rotator of the hip joint. Therefore, whenever the piriformis muscle is irritated or inflamed, it also affects the sciatic nerve, resulting in sciatic pain.^[4]^ The symptoms of PS are similar to those of lumbar disc herniation and lumbar spinal stenosis, which can be easily confused.^[5,6]^ This syndrome is controversial. For its clinical diagnostic characteristics, some people say that it is underdiagnosed^[7,8]^ and some people think that it is overdiagnosed.^[9]^ Therefore, in this case, we need to clarify its main clinical features to improve the accuracy of clinical diagnosis. Studies have shown that the most common clinical features of piriformis syndrome are: buttock pain, pain aggravated on sitting, external tenderness near the greater sciatic notch and pain on any maneuver that increases piriformis muscle tension, and limitation of straight leg raisin.^[10]^ Therefore, in clinical diagnosis, the diagnosis should be distinguished strictly to reduce the misdiagnosis rate. The traditional treatment of this disease is mainly oral administration of diclofenac sodium or ibuprofen and other antipyretic, analgesic, and anti-inflammatory drugs, but its therapeutic effect is not good and the adverse reactions are large. Some patients even experienced complications such as gastric bleeding after oral medication. Even if the symptoms are controlled for a period of time, the possibility of relapse after stopping the drug is high. At the same time, the routine surgery of lysis can cause severe trauma and bleeding to the patient. After the operation, the loosened tissue will re-adhesion and even worsen symptoms. However, the treatment of piriformis syndrome with small needle knife lysis solves the problem of piriformis compression of the sciatic nerve, and the Little needle-scalpel is a steel object with a certain degree of toughness and less damage to the piriformis muscle.^[11]^

### 1.2 Description of the intervention

Little needle-scalpel is a kind of acupuncture tool made of metal material that resembles a needle and a knife in shape. It is developed on the basis of the ancient 9-needle needles, sharp needles, etc, combined with modern medical surgical scalpels, and is also a product of organic integration with soft tissue release surgery. Little needle-scalpel therapy is a closed lysis between surgical therapy and non-surgical therapy. Its theoretical basis is the theory of dynamic stability and imbalance, and the pathological basis is soft tissue adhesions, scars, and contractures.^[12]^ It is mainly suitable for soft tissue lesions and bone and joint diseases, to penetrate deep into the lesion for easy cutting, peeling, etc, in order to achieve the purpose of pain relief and disease removal.^[13]^

### 1.3 How the intervention might work

Based on the pathological mechanism of piriformis injury, the main measures to treat the disease are to solve the congestion, swelling, spasm, scarring, and adhesion after injury. Little needle-scalpel therapy is an ideal therapy for peeling adhesions and dredging blockages. On the one hand, the loosening and stripping of the needle knife can strengthen the vascular permeability around the piriformis muscle, improve blood circulation, relieve spasm pain, and accelerate the resolution of secondary inflammatory reactions such as congestion and edema. On the other hand, the piriformis can be cut and smoothed by cutting and guiding the piriformis muscle, peeling the adhesion, and directly eliminating the swelling. This can reduce or eliminate the compression and stimulation of the blood vessels and nerves by the lesion, so as to achieve the purpose of adhesion peeling, blocking dredging, and smoothing of blood and blood.^[14]^

### 1.4 Why it is important to do this review

For patients with compressed nerves, massage, acupuncture, and local block are generally used clinically.^[15,16]^ However, this type of treatment is long and prone to relapse, so many patients will eventually need surgery. Traditional open surgery is more traumatic, prolongs the patient's recovery time, and causes considerable trauma to the patient's physiology and psychology. Little needle-scalpel therapy draws on the strengths of traditional Chinese acupuncture therapy and Western surgical therapy, organically combines the 2 methods, and has a unique effect on the adhesion of chronic soft tissue injuries and the sequelae of partial bone and joint injuries. It not only overcomes the shortcomings of Western medicine such as large damage, greater sequelae, and scars on the skin, but also has the curative effect of traditional Chinese medicine acupuncture.^[17]^ In view of the small damage to the hip muscles by the small needle knife and the quick recovery of the patient, it has been recognized by clinicians.^[18]^

### 1.5 Objectives

To systematically evaluate the effectiveness and safety of Little needle-scalpel therapy for piriformis syndrome patients.

## 2 Methods and analysis

This protocol was designed in accordance with the methodological guidelines for overviews provided by the Cochrane Handbook for Systematic Reviews of Interventions.^[19]^ It is registered on the International Prospective Register of Systematic Reviews. (Registration number INPLASY2020110092; https://inplasy.com/inplasy-2020-11-0092/.)

### 2.1 Inclusion criteria for study selection

#### 2.1.1 Types of studies

Randomized controlled trials (RCTs) will be included, without restrictions on publication status.

#### 2.1.2 Types of participants

Adult patients with piriformis syndrome, regardless of sex, race, or educational and economic status.

#### 2.1.3 Types of interventions

Research using Little needle-scalpel or combined with other therapies, without limiting the treatment time and dose.

#### 2.1.4 Types of comparisons

The control group's treatment is not limited, including no treatment, placebo, or any control considered for comparison in a single systematic review.

#### 2.1.5 Types of outcome measures

Primary outcomes:

1. Clinical symptoms and signs.
2. Physical activity.

## 3 Clinical efficacy

### 3.1 Search methods for identification of studies

#### 3.1.1 Electronic searches

The following databases from the inception to December 2020 will be searched by 2 independent reviewers, without restriction to publication status and languages: the Cochrane Central Register of Controlled Trials (CENTRAL); PubMed; EMBASE; China National Knowledge Infrastructure (CNKI); Chinese Biomedical Literature Database (CBM); Chinese Scientific Journal Database (VIP database); and Wan-Fang Database. A search strategy for PubMed database, which is established according to the Cochrane handbook guidelines, is shown in Table 1. Similar search strategies will be applied for the other databases. Before this review completed, the 2 reviewers will conduct the searching once again to ensure the latest studies could be included.

**Table 1:** Search strategy for the PubMed database.

Number	Search terms
1	Piriformis syndrome
2	Musculi piriformis syndrome
3	or 1–2
4	Little needle-scalpel
5	Acupotomy
6	Knife-needle
7	or 4–6
8	3 and 7

#### 3.1.2 Searching other resources

Besides, electronic sources for relevant researches in progress will also be searched, including Clinicaltrials.gov (http://www.clinicaltrials.gov) and the World Health Organization International clinical trials registry search portal (http://apps.who.int/trialsearch/). Additionally, the citation list will be retrieved in Web of Science. Besides, the reference lists of those studies meeting the inclusion criteria and relevant systematic reviews will also be identified for additional relevant studies.

### 3.2 Data collection and analysis

#### 3.2.1 Selection of studies

**Figure 1:**
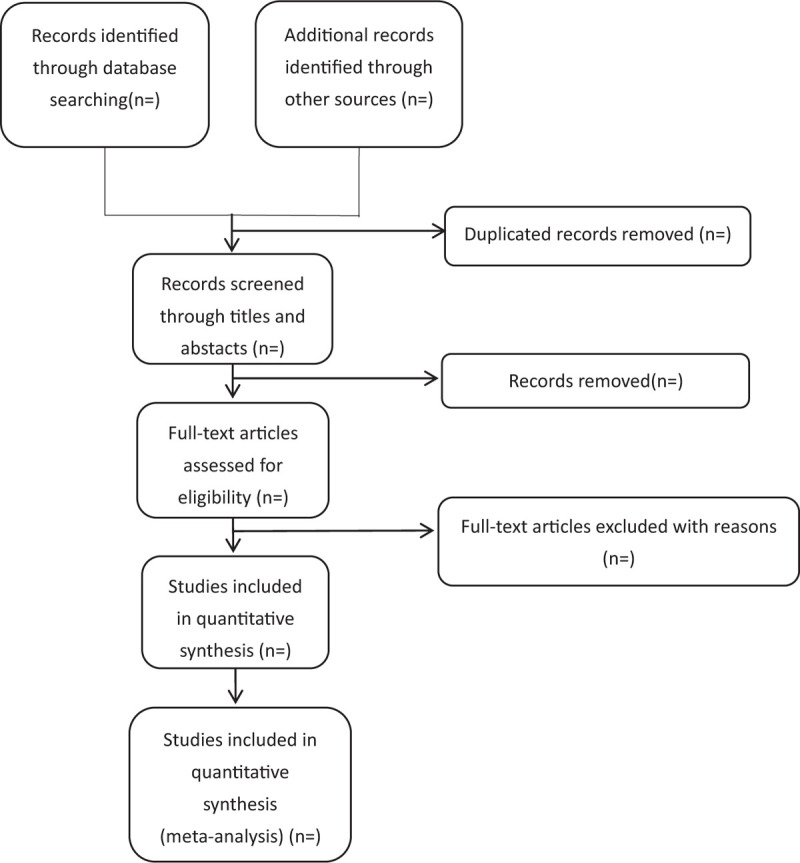
Flowchart of literature selection.

We plan to conduct this systematic review between December 30, 2020 and July 30, 2022. All reviewers have undergone a training to ensure a basic understanding of the background and purpose of the review. After electronic searching, the records will be uploaded to a database set up by EndNote software (V.X7), Thomson ResearchSoft, US. Records selected from other sources will also be moved to the same database. Two reviewers (SYZ and GHT) will independently screen the titles, abstracts, and keywords of all retrieved studies and decide which trials meet the inclusion criteria. We will obtain the full text of all possibly relevant studies for further assessment. Excluded studies will be recorded with explanations. Any disagreements will be resolved by discussion between the 2 reviewers (SYZ and GHT) and the third author (JC) for arbitration when necessary. We will contact reviewers of trials for clarification when necessary. The study flow diagram is shown in Fig. 1.

### 3.3 Data extraction and management

A unified data extraction form will be designed by all of the reviewers and its applicability will be tested in a small scope of trials by 2 reviewers (ZYZ and LBL). They will then independently extract data in the following domains: general information, participants, methods, interventions, outcomes, results, and other information. Any disagreement will be discussed between the 2 reviewers, and further disagreements will be arbitrated by the third author (JC).

### 3.4 Assessment of risk of bias in included studies

The risk of bias will be assessed by 2 reviewers (QYZ and SYZ) with the Cochrane Collaboration's tool for risk of bias assessment. The risk of bias in included studies will be evaluated according to the following aspects: sequence generation, allocation sequence concealment, blinding of participants and personnel and outcome assessors, incomplete outcome data, selective outcome reporting, and other sources of bias. The assessments will be classified into 3 levels: low risk, high risk, and unclear risk.

### 3.5 Measures of treatment effect

RevMan V.5.4 (Cochrane Collaboration) will be used for data analysis and quantitative data synthesis. For continuous data, we will use standard mean difference (SMD) to measure the treatment effect with 95% confidence intervals (CIs). For dichotomous data, a risk ratio (RR) with 95% CIs for analysis will be adopted.

### 3.6 Unit of analysis issues

Data from studies with parallel-group will be included for meta-analysis. For randomized cross-over trials, only the first phase data will be included. In these trials, participants are individually randomized to 2 intervention groups, and a single measurement of each outcome from each participant is collected and analyzed.

### 3.7 Dealing with missing data

We will try to contact the first or corresponding authors of the included studies by telephone or email to retrieve missing or insufficient trial data. If missing data are unavailable, we will make an assumption using the terms “missing at random” and “not missing at random” to represent different scenarios, which is recommended in the Cochrane Handbook.^[20]^ For the data “missing at random,” only the available data will be analyzed. For the data “not missing at random,” we will displace the missing data with replacement values and a sensitivity analysis will be used to determine whether the results are inconsistent.

### 3.8 Assessment of heterogeneity

On the basis of the data analysis, random effect or fixed effect models will be employed according to the heterogeneity given by *I*^*2*^ statistic value. To be concrete, a fixed effect model will be adopted if the heterogeneity is indicated as high (*I*^2^ < 50%); otherwise, a random effect model will be applied on the contrary.

### 3.9 Assessment of reporting biases

We will use funnel plots to detect reporting biases and small study effects. If >10 studies are included in the meta-analysis, we will conduct a test for funnel plot asymmetry using Egger method. All eligible trials will be included, regardless of their methodological quality.

### 3.10 Data synthesis

The systematic review will be conducted with the use of RevMan 5.4. Taking account of the heterogeneity assessment, mean difference or relative risk (RR) with fixed or random effect model will be computed. Additionally, if heterogeneity is considered significant, the sensitivity or subgroup analysis will be generated to distinguish the source of it. When it comes to the situation that the data are insufficient for quantitative analysis, the review will only represent and summarize the evidence.

### 3.11 Sensitivity analysis

Sensitivity analysis will be conducted to validate the robustness of the primary results. We will exclude certain trials by a reevaluation of methodological quality, study types, sample size, missing data, or other possible factors. Careful interpretations will be employed for sensitivity analysis if differ substantially.

### 3.12 Grading the quality of evidence

The Grading of Recommendations Assessment, Development and Evaluation (GRADE) working group methodology will be applied for the quality of evidence for all outcomes.^[21]^ Six domains will be assessed, containing risk of bias, consistency, directness, precision, publication bias, and additional points. The assessments will be categorized into 4 levels: high, moderate, low, or very low.

### 3.13 Subgroup analysis

If data are available, a subgroup analysis will be performed based on patient baseline characteristics, control mode, and so on. Because this is the main factor causing heterogeneity.

## 4 Discussion

The treatment of piriformis syndrome generally includes short-term rest (not >48 hours), the use of muscle relaxants, non-steroidal anti-inflammatory drugs, and physical therapy (including piriformis stretching, activity, and deep tissue massage). For some patients, injecting steroids around the piriformis muscle may help reduce inflammation and pain.^[22]^ Surgery is the last consideration for patients with piriformis syndrome. Because the results after surgery are not always predictable, and some patients still feel pain.^[23]^ The small needle knife not only has the function of acupuncture in traditional Chinese medicine, but also has the releasing function of western medicine surgery.^[24]^ Using a small needle knife to act on the strong stimulation of the human body can directly loosen the fascia tissue of the adhesion contracture, restore the dynamic balance of the muscle, relieve the pain of spasm, and strengthen the vascular permeability around the piriformis muscle.^[25]^ Therefore, it is very meaningful to explore the effectiveness and safety of Little needle-scalpel in the treatment of piriformis syndrome.

## Author contributions

**Conceptualization:** Qingyuan Zhu, Jun Xiong.

**Data curation:** Qingyuan Zhu, Siyuan Zhu, Lunbin Lu, Jun Chen, Zhiying Zhong, Genhua Tang.

**Formal analysis:** Siyuan Zhu, Jun Chen.

**Investigation:** Qingyuan Zhu, Jun Xiong, Zhiying Zhong.

**Methodology:** Siyuan Zhu, Lunbin Lu, Jun Chen.

**Software:** Siyuan Zhu, Jun Chen.

**Supervision:** Jun Xiong, Lunbin Lu.

**Writing – original draft:** Qingyuan Zhu, Jun Xiong.

**Writing – review & editing:** Jun Xiong, Lunbin Lu, Jun Chen, Genhua Tang.
